# Assessment of community pharmacy professionals’ willingness, involvement, beliefs, and barriers to offer health promotion services: a cross-sectional study

**DOI:** 10.1186/s12913-022-08944-w

**Published:** 2022-12-17

**Authors:** Wondim Ayenew, Abdulwase Mohammed Seid, Asmamaw Emagn Kasahun, Asrat Elias Ergena, Derso Teju Geremaw, Liknaw Workie Limenh, Teshome Bitew Demelash, Wudneh Simegn, Yeniewa Kerie Anagaw

**Affiliations:** 1 Department of Social and Administrative Pharmacy, University of Gondar, Gondar, Ethiopia; 2 Department of Clinical Pharmacy, University of Gondar, Gondar, Ethiopia; 3 Department of Pharmaceutics, University of Gondar, Gondar, Ethiopia; 4 Department of Pharmaceutical Chemistry, University of Gondar, Gondar, Ethiopia; 5 Department of Pharmacy, Pawi Health Science College, Pawi, Ethiopia

**Keywords:** Community pharmacy professionals, Community pharmacy, Health promotion, Injibara

## Abstract

**Introduction:**

The role of community pharmacy professionals has been expanded to patient care and health promotion service globally. However, in Ethiopia, there is a scanty of data on the issue, although the country is dealing with a double burden of non-communicable and communicable diseases.

**Objectives:**

This study aimed to assess community pharmacy professionals’ willingness, involvement, beliefs, and barriers to offer extended services for health promotion in Injibara town, Amhara, Ethiopia.

**Methods:**

A cross-sectional study was conducted among licensed and registered community pharmacy professionals working in Injibara town from June 25 to July 10, 2022. A structured self-administered questionnaire was used to collect data. The data were presented using descriptive statistics. The data were analyzed using STATA version 16 software.

**Results:**

A total of 24 community pharmacy professionals were involved in the study, with a response rate of 92.3%. Approximately 91.7% of them were involved in health promotional services. Of them, 54.1% were willing and strongly believed that their involvement in health promotion services would have a positive impact on promoting health. A total of 60.9% of the community pharmacy professionals reported that they were very involved in family planning and alcohol consumption counseling. Different barriers to not providing health promotion services were also cited.

**Conclusions:**

Majority of community pharmacy professionals in this study is involved in health promotional services but there are also barriers on their involvement. Therefore, governmental strategies to overcome the barriers that hamper their involvement should be designed.

## Introduction

Globally, the role of community pharmacy professionals is shifting from drug compounding and dispensing to patient care and health promotion [1]. Health promotion is the process of empowering people to exert more control over and improve their health [2]. Instead of concentrating on those who are at high risk for particular diseases, this involves the population as a whole in the context of their daily lives [3]. To combat health risks, health promotion combines a variety of methods, all of which are complementary to one another. These methods include communication, education, legislation, financial incentives, organizational change, community development, and impromptu local activities. It is a guiding principle that involves actions meant to improve both individual and communal health and well-being [2, 3].

Health promotion is now recognized as a crucial component of contemporary pharmacy practice due to changes in pharmacy practice across the globe [4, 5]. Community pharmacy professionals can improve patients’ outcomes and lower healthcare costs through health promotion [6]. They are frequently cited as the most approachable of all medical professionals and are frequently patients’ first and only point of contact with the healthcare system [7].

Currently, community pharmacy professionals offer and are involved in health promotion services, including therapeutic medication management and patient counseling [8], cessation of smoking [9], diabetes mellitus [10, 11], hypertension [12, 13], dyslipidemia [12], contraception [14], asthma [15] and immunization [16, 17]. Moreover, they gave consultation for a variety of health risks and conditions, including diabetes, smoking cessation, weight management, hypertension, osteoporosis, and substance abuse. Mostly, their advice and general information’s distribution were in the form of verbal communication in response to symptoms and during the sale of over-the-counter medicines and the distribution of general information [4, 18].

Despite the potential involvement of community pharmacy professionals in public health promotion, the provision of public healthcare promotion services in community pharmacy settings has been hampered by a number of obstacles, including a lack of knowledge and skills, confidence, adequate training and policies, poor recognition within the health care system, patients’ reluctance to use pharmacy services, and the presence of an insufficient number of pharmacy staff [19, 20].

Community pharmacy professionals in Ethiopia, such as druggists and pharmacists, are rarely involved in health promotion services, although the country is dealing with a double burden of non-communicable and communicable diseases [21]. Little is known about how willing community pharmacy professionals in Ethiopia are to participate in public health services. Therefore, this study aimed to assess community pharmacy professionals’ willingness, involvement, beliefs, and barriers to offering extended services for health promotion in Injibara town, Awi zone, Ethiopia.

## Methods

The study was conducted in Injibara, the town of the Awi zone. The Awi zone is one of the 12 zones in the Amhara regional state. There are three district hospitals, one general hospital, 46 health centers and 203 health posts. It also has 190 private health facilities, including 114 clinics, 75 community pharmacies, and one medium diagnostic laboratory center. In urban and rural public health facilities, there are 2043 health professionals and 533 health extension personnel employed [22]. The study was carried out between June 25 and July 10, 2022.

### Study design

A cross-sectional study was conducted among community pharmacy professionals in Injibara town.

### Study population

This study focused and carried out on all licensed and registered community pharmacy professionals who had worked at Inibara town community pharmacies. All community pharmacy professionals who had at least 6 months of professional experience were included in this study. Community pharmacy professionals who had not worked in Injibara town for the previous 6 months and who refused or did not consent to voluntarily participate in the study were excluded.

### Data collection

Data were gathered by two pharmacists between June 25 and July 10, 2022. Prior to collecting data, the data collectors received training on the aim of the study. The zonal health department provided a list of all the town’s community pharmacies, which were authorized and registered. All community pharmacies were chosen from the list. One community pharmacy professional was assigned to represent each chosen community pharmacy. A structured self-administered questionnaire with slight modification for our needs from earlier studies of a similar nature was developed [23, 24].

Then, 26 community pharmacy professionals were invited, and the questionnaire was distributed to them at their workplace. Six community pharmacy professionals were excluded because they worked in pharmacies established before 6 months. To reduce any potential research bias during the study, the respondents were directly contacted, and the research questionnaires were directly gathered. All of the respondents participated voluntarily and asked for their informed consent to participate in the study. After informed consent was obtained, the authors attested that the participants were aware of the study purpose, risks and benefits. The collected data were kept confidential and only used for the purpose of the study.

The questionnaire consists of a list of questions that evaluates community pharmacy professionals’ willingness, involvement, beliefs, and barriers to participating in health promotion services. Data collectors distributed the English-language questionnaire to all community pharmacy professionals in the town after briefly explaining its purpose. We used a Likert scale (1 = strongly disagree to 5 = strongly agree) to assess the barriers, beliefs, and willingness of community pharmacy professionals to provide health promotion services. With the help of a Likert scale (1 = not involved to 5 = very involved), the level of community pharmacy professionals involvement in the delivery of health promotion services was evaluated.

### Validity and reliability

To ensure the validity of the instrument, data were collected from pharmacy professionals who had more than 6 months of work experience. The data collection instrument was also developed based on literature review with slight modification for our needs from earlier studies of a similar nature and pre-tested by respondents before the study. The reliability of the instrument was tested by using a crobanchs alpha (α). Cronbach’s alpha value greater than 0.70 was considered as acceptable; alpha values greater than 0.8 were considered as good and alpha values greater than 0.9 ware considered as excellent [25]. Cronbach‘s Alpha value was 0.86, indicating acceptable reliability and stability of the instrument (Table 1).

**Table 1:** Cronbach‘s Alpha value of the instrument

Questionnaires	Cronbach’s alpha
Belief and willingness on the importance of providing health promotion	0.79
Barriers to offer health promotion	0.73
Reason for not giving health promotion service	0.90
Requirements that should be fulfilled for to perform health promotional services	0.71
Involvement of community pharmacy professionals in health promotion services	0.88
**Total**	**0.86**

### Data analysis

Before being entered into the computer, the collected data were manually checked for accuracy and consistency. The STATA version 16 program was used to analyses the data. The data were presented using descriptive statistics such as percentage, proportion, and mean. Categorical variables were reported as percentages. Continuous variables were tested for normality by the Shapiro–Wilk test and are presented as the mean with standard deviation (SD) when the data were normally distributed or the median with interquartile range (IQR) when the data were not normally distributed.

**Fig. 1:**
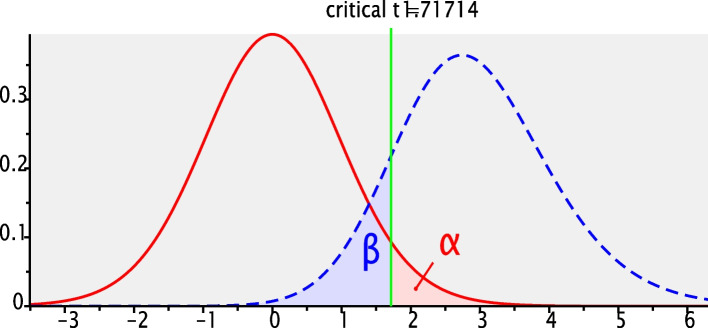
Post-hoc power analysis of the study

Power analysis was performed using G*Power statistical software. A t-test statistic was selected for our study. A post –hoc power analysis (1- β is computed as a function of α, the population effect size, and N) was computed. Based on Cohen’s (1988) power analysis recommendation population effect size is set as 0.5. We want to know the power of the study given α = 0.05 and a total sample size of *N* = 24. The analysis showed that the power of the study was 86.32% (Fig. 1). This indicated that 86.32% of the time we would get a statistically significant result.

**Table:** 

**t tests**	Correlation	Point biserial model
**Analysis**	Post hoc	Compute achieved power
**Input**	Tail(s)	= One
	Effect size |ρ|	= 0.5
	α err prob	= 0.05
	Total sample size	= 24
**Output**	Noncentrality parameter δ	= 2.8284271
	Critical t	= 1.7171444
	Df	= 22
	Power (1-β err prob)	= 0.8632235

## Results

From a total of 32 community pharmacies found in the town of Injibara, a total of 32 community pharmacy professionals worked in the pharmacy. Two community pharmacy professionals did not consent to participate in the research. Six community pharmacies were established before 6 months, and they were excluded from the study. Finally, a total of 24 community pharmacy professionals completed and returned the self-administered questionnaires, for a response rate of 92.3%.

### Socio-demographic characteristics of respondents

The majority of the respondents were males (54.2%). Approximately 62.5% of the respondents were married. The median age of the respondents was 26 years, with an IQR of 24.0–36.5 years. The median experience of the respondents as a community pharmacy professional was 2 years with IQR (1–4.5). Approximately 5 (20.8%) of the respondents had taken in-service job training on health promotion (Table 2).

**Table 2:** Socio-demographic characteristics of the respondents

Socio demographic Variables	n (%)
Gender	
Male	13 (54.2)
Female	11 (45.8)
Age	
20–30	15 (62.5)
31–40	7 (29.2)
> 41	2 (8.3)
Educational status	
Diploma	15 (62.5)
B.Pharm	9 (37.5)
Work experience as community pharmacy professional	
< 5 years	14 (58.3)
5–10 years	7 (29.2)
> 10 years	3 (12.5)
Position in community pharmacy	
Owner	10 (41.7)
Employee	14 (58.3)
On job training on health promotion	
Yes	5 (20.8)
No	19 (79.2)
On Job training obtained on health promotion	
Before [0–2] years	3 (60.0)
Before [3–5] years	1 (20.0)
Before > 6 years	1 (20.0)

### Respondents’ beliefs and willingness regarding the importance of providing public health services

According to the study, the majority of community pharmacy professionals (13, 54.1%) strongly believed that their involvement in health promotion services would have a positive impact on health promotion. The majority of them (54.2%) were also willing to perform health promotion functions (Table 3).

**Table 3:** Belief and willingness of the respondents on the importance of providing public health services

Belief and willingness on the importance of providing public health services	Responses				
	S.A	A	N	D.A	S.D
Health promotion services by community pharmacists make positive change on health outcomes	13 (54.1)	10 (41.7)	0 (0.0)	0 (0.0)	1 (4.2)
Health promotion services by community pharmacists increase quality of life of the community	14 (58.3)	10 (41.2)	0 (0.0)	0 (0.0)	0 (0.0)
Providing health promotional services in community pharmacy will be economical for the community (for the users/client)	11 (45.8)	12 (50.0)	0 (0.0)	1 (4.2)	0 (0.0)
Providing health promotional services in community pharmacy will have positive economic outcome for the community pharmacy	10 (41.7)	13 (54.1)	1 (4.2)	0 (0.0)	0 (0.0)
I am willing to involve on the provision of health promotional activities	13 (54.2)	11 (45.8)	0 (0.0)	0 (0.0)	0 (0.0)
I believe strong health promotional activities can be given by community pharmacists	10 (41.6)	12 (50.0)	1 (4.2)	1 (4.2)	0 (0.0)

*Note*: *SD* Strongly Disagree, *D* Disagree, *A* Agree, *SA* Strongly Agree

### The involvement of community pharmacy professionals in health promotion services

**Fig. 2:**
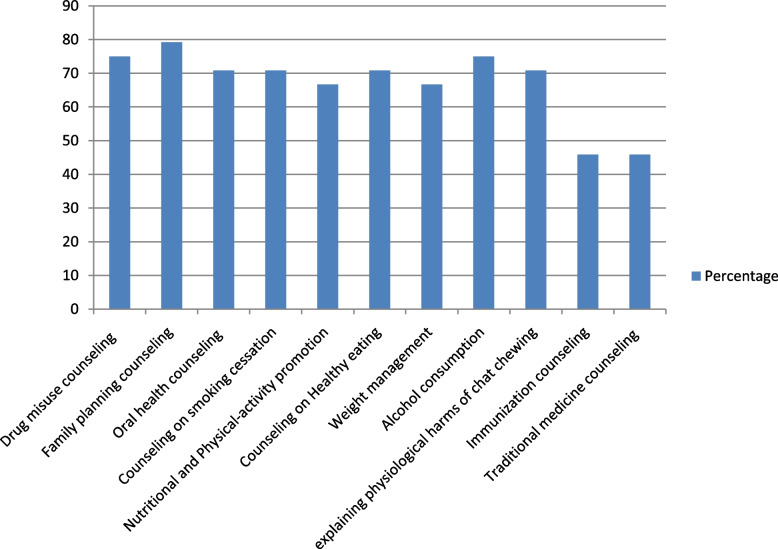
Involvement of community pharmacy professionals in health promotional services

**Fig. 3:**
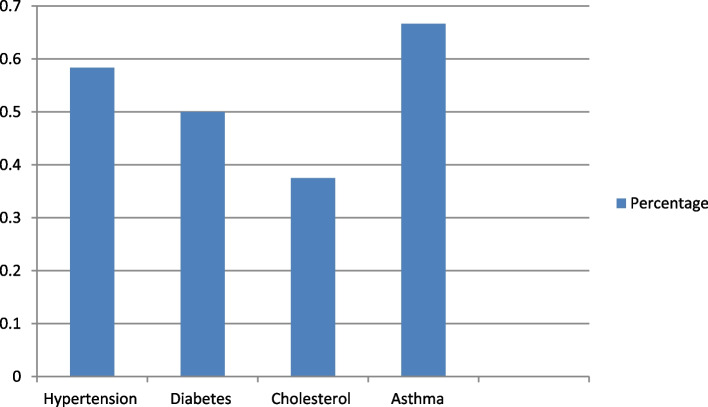
Involvement of community pharmacy professionals in screening of disease

In the previous 6 months, approximately 22 (91.7%) of the respondents were involved in and provided health promotional services in a community pharmacy. The remaining 2 (8.3%) of them were not given any form of health promotion services for the last 6 months. The majority of respondents (79.2%) provided family planning counseling in the community pharmacies, followed by drug misuse and alcohol consumption counseling (75% each). Immunization counseling (45.8%) and traditional medicine counseling (45.8%) were the least common services provided by community pharmacy professionals. Asthma screening service (66.7%) was the major screening service provided, followed by hypertension (58.0%) in community pharmacies for the last 6 months (Figs. 2 and 3).

The proportion of community pharmacy professionals who reported their community pharmacy as being very involved in each service was 14 (60.9%) for involvement in family planning counseling and alcohol consumption counseling (Table 4).

**Table 4:** The level of involvement of community pharmacy professionals in health promotion services

Involvement in health promotion	Responses n (%)				
	Very Involved	Involved	Moderate Involved	Slightly Involved	Not Involved
**Behavioral and Life style modification**					
Drug misuse counseling	13 (56.5)	9 (39.1)	1 (4.4)	0 (0.0)	0 (0.0)
Family planning counseling	14 (60.9)	8 (34.7)	1 (4.4)	0 (0.0)	0 (0.0)
Oral health counseling	10 (43.5)	8 (34.8)	3 (13.0)	2 (8.7)	0 (0.0)
Counseling on smoking cessation	9 (39.1)	10 (43.5)	2 (8.7)	2 (8.7)	0 (0.0)
Nutritional and Physical-activity promotion	7 (30.4)	13 (56.5)	2 (8.7)	1 (4.4)	0 (0.0)
Counseling on Healthy eating	7 (30.4)	13 (56.5)	2 (8.7)	1 (4.4)	0 (0.0)
Weight management	3 (13.0)	14 (60.8)	4 (17.4)	1 (4.4)	1 (4.4)
Alcohol consumption	14 (60.9)	7 (30.4)	0 (0.0)	1 (4.4)	1 (4.4)
explaining physiological harms of chat chewing	6 (26.0)	8 (34.8)	8 (34.8)	1 (4.4)	0 (0.0)
Immunization counseling	5 (21.7)	10 (43.5)	7 (30.4)	1 (4.4)	0 (0.0)
Traditional medicine counseling	2 (8.7)	9 (39.1)	4 (17.4)	4 (17.4)	4 (17.4)
**Screening**					
Hypertension	8 (34.8)	1 (4.4)	6 (26.0)	6 (26.0)	2 (8.7)
Diabetes	7 (30.4)	1 (4.4)	2 (8.7)	11 (47.8)	2 (8.7)
Cholesterol	1 (4.4)	3 (13.0)	1 (4.4)	12 (52.1)	6 (26.1)
Asthma	5 (21.7)	2 (8.7)	2 (4.4)	12 (52.2)	3 (13.0)

### Barriers to providing health promotion services in community pharmacies

There were different barriers to not providing health promotion services. Lack of knowledge, lack of time, lack of confidence, lack of on-the-job training, employers’ unwillingness to pay more if the pharmacist’s scope of practice is wider, the space is not enough to perform promotional activity, insufficient management support, regulatory bodies do not allow to do so, and absence of standard guidelines for the service were indicated as barriers to rendering health promotion services in community pharmacies.

### Opinion of pharmacy professionals on the requirements fully fulfilled to perform health promotion services

Community pharmacy professionals list the requirements that should be fulfilled to perform health promotional services in community pharmacies. Approximately 95.8% of the respondents said that providing in-service trainings for community pharmacy professionals to perform health promotional services is an important criterion to be fulfilled. The respondents also cited the following requirements to perform health promotional activities in community pharmacies: make more payments for pharmacists (19 (79.2%)), arrange private space for the client and the pharmacist (9 (37.5%)), add different courses to the curriculum to provide health promotional functions (10 (41.7%)), and increase the number of pharmacy professionals (14 (58.3%)).

## Discussion

This study aimed to assess community pharmacy professionals’ willingness, involvement, beliefs, and barriers to offer health promotion services. The majority of the community pharmacy professionals were involved in health promotional services, and more than half of them were willing and had a strong belief that their involvement in health promotion services would have a positive change in promoting health. They were more involved in family planning, alcohol consumption counseling and asthma screening. Even though their involvement is great, they encounter different barriers to providing health promotion services.

Pharmacy practice has evolved with extended roles in patient care and providing medicine use information for the community. Community pharmacy professionals now have expanded the roles of preventive and holistic care to the community. The easy access of community pharmacy professionals to patients has positioned the profession towards the incorporation of advanced health promotion activities. The active participation of community pharmacy professionals in health promotion may serve as a needed link in the sustained global push towards providing increased access to essential medicines, especially in developing communities, and it will improve overall health coverage [1].

In the present study, the majority of community pharmacy professionals strongly believed that their involvement in health promotion services would have a positive change in promoting health. This study is consistent with a study performed in Canada that showed a positive attitude of community pharmacy professionals in health promotion, especially in the area of health screening for hypertension, diabetes, dyslipidemia, smoking cessation, sexual health, infectious disease control, and immunization [26].

The study also agreed with a study done in Nigeria in which community pharmacy professionals indicated a favorable attitude towards health promotion services and showed improvement in the services offered in their community pharmacies. Studies conducted in Canada [26], Rwanda [27] and Nigeria [28] showed that community pharmacy professionals are willing to provide health promotion services. This is consistent with our study, in which community pharmacists were willing to perform health promotion functions. The majority of community pharmacy professionals are willing to perform health promotion functions in community pharmacies; they also believe that providing health promotional services in community pharmacies has positive economic and health outcomes.

Community pharmacy professionals engage in a variety of health promotion services, such as counseling patients on behavioral and lifestyle changes and screening them for chronic illness problems [8–13, 15]. Giving information to caregivers about the options for disease management or prevention fosters positive behavioral change in caregivers. There is information available regarding other policy areas that promote health, such as monitoring illnesses and working conditions, avoiding overcrowding, using medications, having a suitable housing situation, and self-care. These services enable people and communities to assume control over the modifiable determinants of health. Supporting, promoting, and diversifying these initiatives while keeping them ongoing as a process will retain the beneficial results and have a significant impact on community health. These activities encourage people to move to a state of optimal health, which is a balance between physical, emotional, social, spiritual, and intellectual health [29, 30].

Pharmacy professionals are involved in different chronic disease screening for hypertension, dyslipidemia, diabetes and asthma and lifestyle counseling in Canada and the USA [26, 31–33]. In the current study, asthma screening services were the major screening service provided, followed by hypertension in community pharmacies, but the provision of screening and counseling services on chronic non-communicable diseases is limited compared to developed countries. This may be due to the structure of health promotion practices, pharmacy laws, and regulations to incorporate health promotion services into community pharmacies, which may differ from country to country. Therefore, community pharmacy professionals should further develop their health promotion service in chronic disease screening to successfully tap into their significant contribution to patient care.

In this study, approximately 91.7% of community pharmacy professionals were involved and rendered health promotional services in community pharmacies. The majority of them (79.2%) provided family planning and alcohol consumption counseling for the communities. A study in South Africa also supported this study and reported that community pharmacy professionals provide family planning services, but it was not free of charge [34].

Studies have also shown the need for community pharmacy professionals to control alcohol consumption. They mentioned the possibility of screening and interventions by community pharmacy professionals to reduce alcoholism in society. They stated that community pharmacy professionals can be effective in the identification of individuals with a high risk of alcohol consumption and then make them aware of the consequences of drinking. Providing alcohol consumption control services in pharmacies can be helpful in reducing the physical and mental health problems of the people. The pharmacists should be trained in communication skills and professional knowledge about alcohol consumption and should provide these services with regard to privacy [35, 36].

In this study, some respondents mentioned different barriers that hinder the full involvement of community pharmacy professionals, including lack of knowledge, lack of time, lack of confidence, lack of on-the-job training, employers’ unwillingness to pay more if the pharmacy professionals’ scope of practice is wider, the space is not enough to perform promotional activities, lack of payment scheme, insufficient management support, regulatory bodies do not allow them to do so, and absence of standard guidelines for the service on community pharmacy professionals discourages participation in health promotion activities. However, community pharmacy professionals would participate more in public health activities if the identified barriers were reduced. They could provide their extended role on health promotion when they receive payment for their work, on-the-job training, and suitable private space for the client and the pharmacist.

This study will assist other researchers to further study the involvement of community pharmacy professionals that can help the healthcare system in adopting strategies to improve community pharmacist participation and to redesign public-private partnerships and the enhancement of accessibility to fundamental healthcare services in neighborhood pharmacies. Our study had some limitations that should be taken into account. Although this study included all community pharmacy professionals, the sample was small, which may affect the generalizability of the results. The study was conducted only in one zonal city, which may also affect the generalizability of the results. We did not use a focus group discussion to arrive at new knowledge. This was the third limitation of our study.

## Conclusions

Community pharmacy professionals working at Injibara community pharmacies are willing and have positive attitudes to perform health promotion services in community pharmacies and strongly believe they should play a significant role in health promotion services. Majority of community pharmacy professionals are also involved in health promotional services but there are also barriers on their involvement. Therefore, governmental strategies to overcome the barrier that hamper their involvement should be designed.

## Data Availability

All data generated or analyzed during this study are included in this published article.

## Abbreviations

IQR: Interquartile range
SD: Standard deviation
